# MD-SVM: a novel SVM-based algorithm for the motif discovery of transcription factor binding sites

**DOI:** 10.1186/s12859-019-2735-3

**Published:** 2019-05-01

**Authors:** Jialu Hu, Jingru Wang, Jianan Lin, Tianwei Liu, Yuanke Zhong, Jie Liu, Yan Zheng, Yiqun Gao, Junhao He, Xuequn Shang

**Affiliations:** 1 School of Computer Science, Northwestern Polytechnical University, West Youyi Road 127, Xi’an, 710072 China; 2 Centre of Multidisciplinary Convergence Computing, School of Computer Science, Northwestern Polytechnical University, 1 Dong Xiang Road, Xi’an, 710129 China

**Keywords:** Transcription factor, Binding site preference, Multiple instance learning, Support vector machine

## Abstract

**Background:**

Transcription factors (TFs) play important roles in the regulation of gene expression. They can activate or block transcription of downstream genes in a manner of binding to specific genomic sequences. Therefore, motif discovery of these binding preference patterns is of central significance in the understanding of molecular regulation mechanism. Many algorithms have been proposed for the identification of transcription factor binding sites. However, it remains a challengeable problem.

**Results:**

Here, we proposed a novel motif discovery algorithm based on support vector machine (MD-SVM) to learn a discriminative model for TF binding sites. MD-SVM firstly obtains position weight matrix (PWM) from a set of training datasets. Then it translates the MD problem into a computational framework of multiple instance learning (MIL). It was applied to several real biological datasets. Results show that our algorithm outperforms MI-SVM in terms of both accuracy and specificity.

**Conclusions:**

In this paper, we modeled the TF motif discovery problem as a MIL optimization problem. The SVM algorithm was adapted to discriminate positive and negative bags of instances. Compared to other svm-based algorithms, MD-SVM show its superiority over its competitors in term of ROC AUC. Hopefully, it could be of benefit to the research community in the understanding of molecular functions of DNA functional elements and transcription factors.

## Introduction

Protein-DNA interactions play essential roles in the regulation of gene transcription, splicing, translation and degradation. The binding of transcription factors (TFs) and DNA is a fundamental molecular mechanism in gene regulation. Gene expression is dynamically regulated by TFs through sequence-specific interactions with genomic DNA. Interactions of TF and DNA binding sites can prevent transcription of downstream genes or activate it. It’s common to see that some genes are co-expressed in specific tissues or during specific cell stage. It indicates that they may be controlled by a common TF regulator. Binding regions of one transcription factor on different genes are usually conservative. The identification of transcription factor binding sites, also known as motif discovery (MD) problems, is usually defined as finding similar subsequences from a given set of DNA sequences [1]. Thus, the accurate characterization of TF-DNA binding affinities is of significance for a quantitative understanding of cellular regulation mechanism in life processes.

In early bioinformatics, the recognition of transcription factor binding sites was mainly concentrated in promoter regions. Many computational tools were developed to uncover the biological function of these functional element using various models [2–11]. In recent years, with the development of high-throughput sequencing technologies, the scope of research has been extended to whole genomes by specific protein and specific DNA sequences of immunoprecipitation throughout entire genomes. In addition, protein binding microarrays (PBM) can be used to measure in vitro transcription factor binding through the array of exhaustive short amino acid sequences on microarrays [12]. Since the common confounding factor was eliminated in the ChIP-Seq experiment [13], PBM data conveyed perfect information in a more direct manner for the modeling of transcription factor binding sites [14].

Recent advances in biotechnologies, such as ChIP-seq, in-vitro protein binding microarrays (PBMs), in-vitro high-througput sequencing and bacterial one-hybrid assays, have provided opportunities to learn sequence motifs of transcription factors using data-driven approaches. The PBM technology enables the rapid, high-throughput characterization of the sequence specificities of DNA-protein interactions in vitro [15]. Many computational approaches have been developed to predict protein binding affinities from PBM data. Position weight matrices (PWMs) are commonly used to characterize binding affinity between TFs and DNA sequences [16–18]. In PWMs, there is a *D*×*L* matrix representing the binding preference of a TF, where *D* is the number of alphabet (4 for DNA sequences), *L* is the length of binding sequences. Given a sequence **x**:=(*x*_1_,*x*_2_,⋯,*x*_*L*_), a log-odds score $S(\mathbf {x})=\sum _{j=1}^{L}\log _{2}(p_{j}(x_{j})/p_{bg}(x_{j}))$ was calculated to indict the binding affinity of **x** with a specific TF [19]. In the formula, *p*_*j*_(*x*_*j*_) is the probablity of nucleotide *x*_*j*_ at the position *j* of the binding site, and *p*_*bg*_(*x*_*j*_) is the background probability of *x*_*j*_ in a representative sequences [20].

Each nucleotide is independent of nucleotides at other positions in this binding sequence. PWMs of thousands of transcript factors are publicly available in motif datasets such as JASPAR [21, 22], TRANSFAC [23, 24].

In contrast to PWMs, nucleotide dependence has been taken into consideration in some statistical models to improve the prediction of binding affinities. A discriminative learning method based on hidden markov model was applied to discover motifs from a variety of high-throughput technologies, including ChIP-Seq [25, 26], RIP-Chip [27, 28] and PAR-CLIP [29, 30] of transcript factors and RNA binding proteins. A Bayesian Markov model (BaMM) was proposed to discover motif, which learns the *k*th-order probability $p_{j}^{(k)}(x_{j}|x_{j-k:j-1})$ using the order-(k-1) probability $p_{j}^{(k-1)}(x_{j}|x_{j-k+1:j-1})$ as prior information [19]. However, the prediction of binding specificity of most eukaryotic TFs remains a challenging problem.

To prevent overtraining, we proposed a novel discriminative algorithm for motif discovery based on support vector machines, which was referred to MD-SVM. It tries to learn an appropriate nonlinear model from training datasets. Basically, there are three major steps in the MD-SVM approach. Firstly, it translates the MD problem into a computational problem of multiple instance learning (MIL), which models each input sequence as a labeled bag with a set of instances [31, 32]. Then, the structure information of each instance (a fragment) was mapped to a feature vector using a nonlinear model. Lastly, a SVM-based method was applied to find an appropriate classifier using the gaussian kernel on a set of training datasets.

## Methods

### Multiple instance learning

The problem of multiple instance learning is to learn a model, which can distinguish a set of given positive and negative bags of instances. Each bag contains many instances. It assumes that a bag is positive only if it has at least one positive instance, and all instances in a negative bag are negative. Given *m* bags *B*_1_,*B*_2_,...*B*_*m*_, there are *k*_*i*_ instances in each bag *B*_*i*_, 1≤*i*≤*m*. There is a label for each bag. Without loss of generality, each bag *B*_*I*_ has a label *Y*_*I*_∈{−1,1}. According to the definition of MIL, if the label of a bag is positive, the bag contains at least one positive instance. If the label of a bag is negative, the labels of all instances in the bag are negative. It can be written into the following formula:

1 $$ \sum_{i\in I}\frac{y_{i}+1}{2}\geq 1,\forall I \ s.t. \ Y_{I} = 1  $$

2 $$ y_{i} = -1, \forall I \ s.t. \ Y_{I} = -1  $$

MIL model has been applied to predict whether a drug molecule will strongly bind to a target protein, which is known to be involved in some diseases. Here, we attempt to solve the MD problem in the framework of MIL. The major task of a MD problem is to find binding preference of a target transcription factor.

### Instance feature extraction

We have modeled the motif discovery problem as a multiple instance learning model problem. However, in the multiple instance learning model, each instance in the bag needs to be converted to a corresponding feature. Hence, it is necessary to convert the sequence information into numerical features to facilitate the use of multiple instance learning methods. We use a nonlinear model to map the structural information of each instance to a feature vector.

**Fig. 1:**
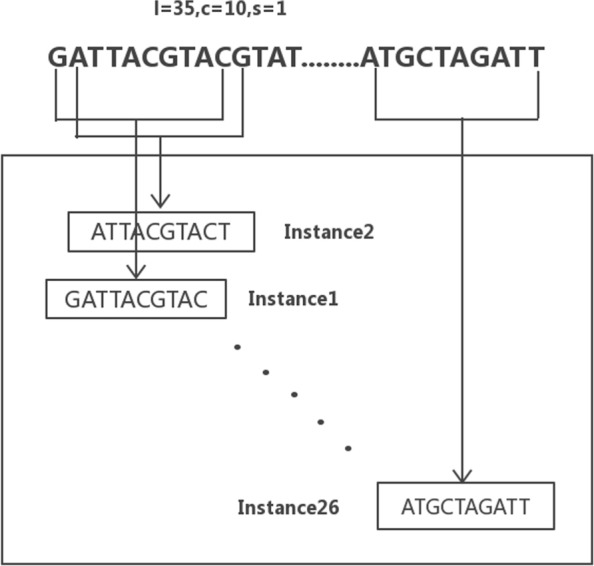
An example of MIL model for DNA fragments

The binding site of transcription factors is generally 5-15 bp in length and conserves in a certain sequence pattern. The probability of a certain base occurring at a certain position may be very high. In the MIL model, we have implicitly scattered all sequences that may be transcription factor binding sites in instances of individual bags. Each probe sequence (*l* =35bp) is considered as a bag in the MIL model. A sliding window (*c* =10bp) was applied to check the substring of each sequence. The sliding window moves forward step by step (*s* =1bp). Then, the instances of each bag would be *n*= [(*l*−*c*)/*s*]+1. Here, *l* is the length of a probe sequence, *c* the window size, *s* the step size. Each subsequence (an instance) could be a possible binding site of a transcription factor. An example in Fig. 1 shows the framework of MIL model in the prediction of possible binding sites of a transcription factor. In this example, each probe sequence contains *n*=[(35−10)/1]+1=26 instances. The sliding window moves forward till it reaches the last instance, which is ATGCTAGATT. We employed one hot encoding feature to represent the four different nucleotides, which are shown in Table 1. Given one instance of *c* nucleotides, the encoded feature vector is one binary vector with the length of 4∗*c*. In our tests, the parameter of *c* is set to 10. The structure information of each instance was mapped to a feature vector. The motif discovery problem became a computational problem in the multi-instance learning model.

**Table 1:** Binary codes for each nucleotide

Nucleotide	Code
A	(1, 0, 0, 0)
T	(0, 1, 0, 0)
C	(0, 0, 1, 0)
G	(0, 0, 0, 1)

Each nucleotide was encoded in a 4-dimensional vector.

### Motif discovery with MD-SVM

The binary classification method of support vector machines (SVM) was firstly proposed by Vladimir Vapnik et al. in 1992 [33]. It can accurately deal with complex nonlinear boundary models, but usually costs time for the calculation of parameters [34]. It was applied to solve small samples, nonlinear and high dimensional pattern recognition. Here we proposed a multi-instance learning algorithm based on the SVM algorithm, MD-SVM, which is similar to MI-SVM proposed in [35]. Its main subjective is to find a discriminative function which can calculate the instance tags according to given constraints.

In the MIL framework, the label of a bag is determined by the largest instance label in the bag. In the formula 1 and 2, we know that if all the tags in the bag are negative, then the value of $\sum _{i\in I}(y_{i}+1)/{2} = 0$. If $\sum _{i\in I}(y_{i}+1)/{2} = 1$, it means that there is just one tag in the bag that is positive. If $\sum _{i\in I}(y_{i}+1)/{2} > 1$,it means that the tag in the bag has more than one instance is positive. At least one of the tags in the bag is positive when *Y*_*I*_=1.

3 $$ \gamma_{I}\equiv Y_{I} {\underset{i\in I}{\max}}\left(w^{T}x_{i}+b\right)  $$

4 $$ \widehat{Y_{I}} = sgn {\underset{i\in I}{\max}}\left(w^{T}x_{i}+b\right)  $$

In the formula (3), the one with the maximum *w*^*T*^*x*_*i*_+*b* can be considered as the representative instance of a bag. In a positive bag, it would be $\max \limits _{i\in I}\left (w^{T}x_{i}+b\right) > 0$, which indicates that at least one of the tags in this bag is positive. On the contrary, it would be $\max \limits _{i\in I}\left (w^{T}x_{i}+b\right) < 0 $ when a bag is negative. Formula (4) represents the label of this bag. If at least one of the instance in this bag has a positive label, $sgn \max \limits _{i\in I}\left (w^{T}x_{i}+\right) = 1$.On the contrary,$sgn \max \limits _{i\in I}\left (w^{T}x_{i}+b\right) = -1$,the label is positive.

To accurately discriminate all positive bags from the negative ones, it is necessary to make sure that *γ*_*I*_ is far greater than 0 for each bag. From the formulas (3) and (4), we can see that the representative instance of each bag is the one that matters the parameter of our svm model. When the representative instance in each bag is determined, all other instances in all bags become useless for the training of classification. Inspired by this intuition, we define a soft interval classifier for multiple sample learning as belows:

5 $$ {\underset{w,b,\varepsilon}{\min}}\frac{1}{2}\|w\|^{2}+C \sum_{I} \varepsilon_{I}  $$

$$s.t. \ \forall I: \ Y_{I} {\underset{i\in I}{\max}}\left(w^{T}x_{i}+b\right)\geq 1-\varepsilon_{I},\varepsilon_{I} \geq 0. $$

For a negative bag, we can convert the operation with maximization into multiple inequality operations and use the same relaxation factor *ε*_*I*_. Mathematically, it can be written as: *Y*_*I*_=−1, −*w*^*T*^*x*_*i*_−*b*≥1−*ε*_*I*_,∀*i*∈*I*. For a positive bag, we need to introduce a variable *s*(*I*)∈*I*, where *s*(*I*) is the subscript of the representative instance in *B*_*I*_. This allows the constraint to be modified as *w*^*T*^*x*_*s*(*I*)_+*b*≥1−*ε*_*I*_. Hence, the objective function can be modified into the following formula:

6 $$ \ \ {\underset{s}{\min}} {\underset{w,b,\varepsilon}{\min}}\frac{1}{2}\|w\|^{2}+C \sum_{I} \varepsilon_{I}  $$

$$s.t. \forall I:Y_{I} = -1 \ \wedge \ -w^{T}x_{i} - b \geq 1 - \varepsilon_{i}, \ \ \forall i \in I $$

7 $$ or \ Y_{I} = 1 \ \wedge \ w^{T}x_{s(I)} + b \geq 1- \varepsilon_{I}  $$

In the above formula, each positive bag *B*_*I*_ is represented by a representative instance, where *X*_*I*_≡*X*_*s*(*I*)_. Note that all of other instances in the bag (*x*_*i*_,*i*∈*I*∧*i*≠*s*(*I*)) do not contribute to the objective function. For a given selection variable, a double-ended objective function can be derived, which is similar to the standard SVM procedure. Compared to SVM, the main difference is that the constraint parameter *α* is modified to the following form:

8 $$ 0\leq \alpha_{I}\leq C,if \ Y_{I} = 1,then\ 0\leq \sum_{i\in I}\alpha_{i}\leq C  $$

Therefore, each bag is mainly constrained by the parameter *C*. After the calculation of the model parameters *w* and *b*, we use the formula (4) to predict the label of the bag.

The pseudocode of MI_SVM is as Algorithm 1. In the MI-SVM algorithm, as long as the last round of labels (instances of all bags) is identical to the current round of labels, the classifier stops the training and uses the current round of parameters as the final results. It can be applied to the identification of transcription factor binding sites. However, there are limitations in the PBM data of some transcription factors. A lot of false negative bags would be produced in the procedure. In this case, it indicates that the training is not enough and it needs to continue iterating on the tags. Therefore, we propose MD-SVM as an improved version of the MI-SVM algorithm and apply it to identify transcription factor binding sites. In the algorithm of MD-SVM, we use a new criterion to control the iterative loop, which makes the iterative loop converge to a stable state. The pseudocode of MD-SVM algorithm is written in Algorithm 2. The major work is to predict the positive instance of each bag in the test datasets, which can help us obtain the position weight matrix. With the PWM, it is possible to predict the base preference of a transcription factor at each position.


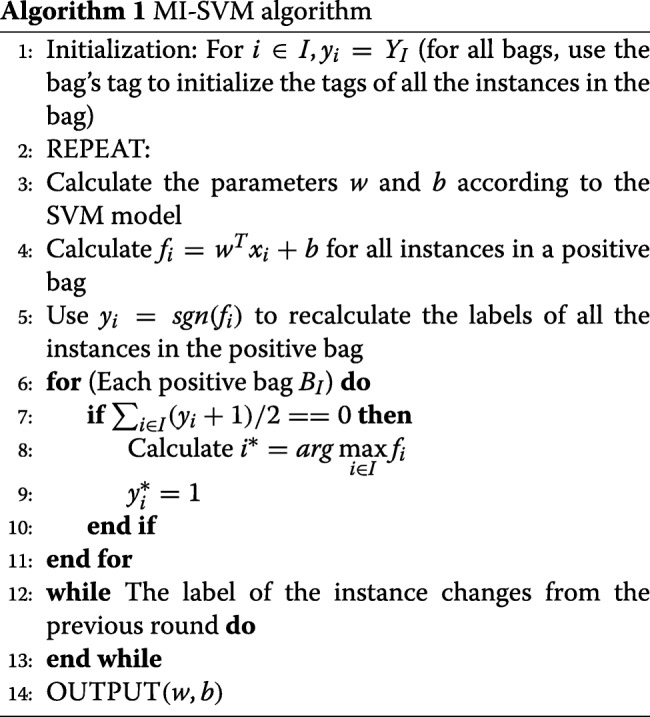



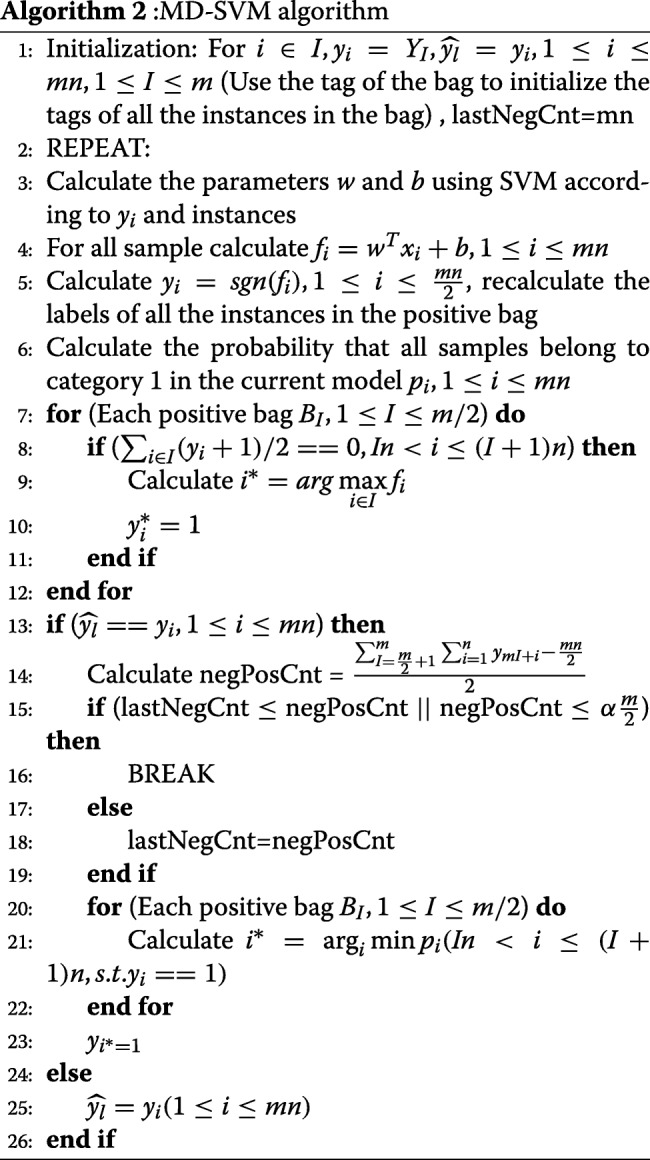


According to the position statistics, the position weight matrix of the transcription factor is obtained, and a seq-logo chart is made, then we observe the base preference of the transcription factor at each position.

## Data and materials

### The preprocessing of PBM data

The PBM technology provides a rapid, high-throughput way to describe the specificity of in vitro binding of transcription factors to DNA. Using microarrays which contains all possible 10-mer sequences, we can obtain TF binding site data for one species. In our experiments, we performed motif discovery algorithms on the PBM data of mice, which was commonly used as test datasets in the DREAM5 challenge (http://dreamchallenges.org). The dataset contains PBM data of transcription factors for a total of 86 mice. The data of each transcription factors were generated from two completely different PBM platforms, HK and ME. Each transcription factor contains two completely different array designs that hybridize the array to different PBM platforms (HK and ME) [36, 37]. Both of the two PBM platforms are designed based on the Agilent 44K array and custom 60bp probes. In each probe, 25 bases were used as flanking sequences. Our test datasets contains 40526 probes in the ME array and 40330 probes in the HK array. These arrays include all possible 10-mer sequence data and 32 repeated non-palindrome 8-mer sequence data, which have no preference for binding of transcription factors. PBM data of one array was used as a training dataset, the other as a test dataset. Since the two datasets are from two different sources, its predictions are more challenging than cross-validations. We performed all our computation on a machine with a 3.1G CPU, 8G memory and a platform of Windows 7 Ultimate 64. A python package sklearn was used as one library in the implication, which is one of commonly used third-party modules.

### Experimental data

Each sequence of the PBM data is in the same length, and is tagged. The top 200 probes with the highest binding strength are used as the positive instances of the training datasets, whereas the last 200 probes used as the negative instances. It can guarantee the reliability of our training datasets, since the binding strength reflect the binding preference of specific sequence.

## Results and Discussion

### Binding preference in sequence logos

**Fig. 2:**
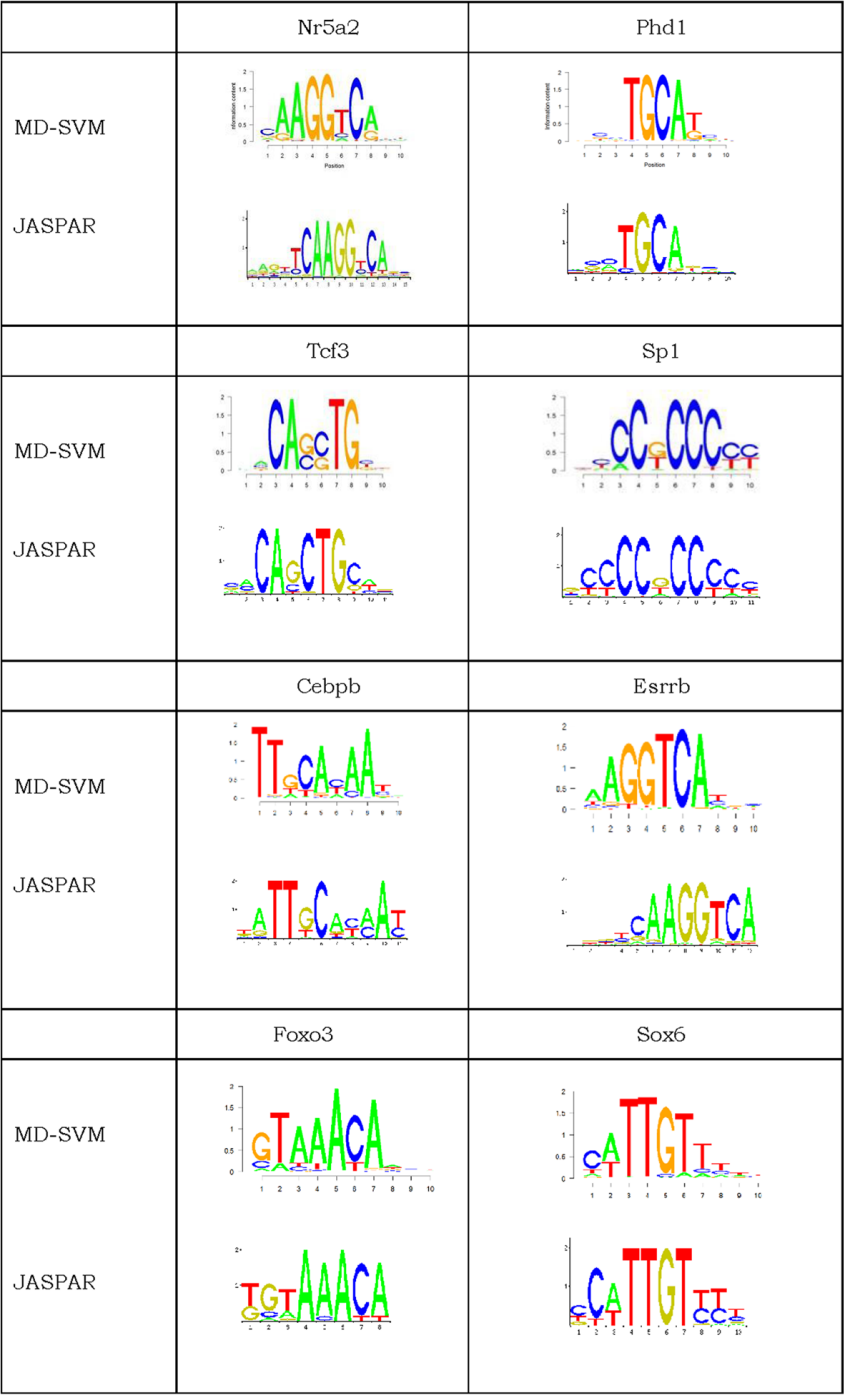
Comparison of motifs discovered by MD-SVM and JASPAR. Here, sequence motifs are graphically displayed in seq-logos. The height of each logo position reflects the degree of sequence conservation in multiple alignments. We compared our seq-logos of eight transcription factors to that extracted from the JASPAR database. Results show that MD-SVM can acurately identify most of the eight transcription factors

Sequence logos are commonly used to show the binding preference of a transcription factor [38]. As shown in Fig. 2, a sequence logo is a graphical display of a multiple sequence alignment consisting of colour-coded stacks of letters representing nucleotides or amino acids at successive positions. The height of a logo position depends on the degree of conservation in the corresponding multiple sequence alignment.

The JASPAR database is a free database containing transcription factor binding site databases of multiple species. To verify the biological quality of the MD-SVM results, we compared the predicted sequence logos to that of the JASPAR database. For instance, both of the sequence logos show that the binding sites of Foxo3 is preferred to be a DNA fragment containing GTAAACA. These conserved patterns in JASPAR were also identified by our method MD-SVM. This shows that our algorithm is advantageous in terms of motif discovery. The motifs identified by MD-SVM is shown in Fig. 2.

We performed MD-SVM and MI-SVM on the test datasets for 18 transcription factors. From Fig. 2, we can see that most of the predicted sequence logos have the same pattern as that of JASPAR reference databases. For instance, both of the sequence logos show that the binding sites of Foxo3 is preferred to be a DNA fragment containing GTAAACA. These conserved patterns in JASPAR were also identified by our method MD-SVM.

### Performance comparison with MI-SVM

The ROC curve is a graphical plot that illustrates the diagnostic ability of tested algorithms. To evaluate the performance of MI-SVM and MD-SVM, we used a measure ROC AUC (area under curve), which is commonly used in the evaluation of binary classifier systems. Fore each threshold, the value of AUC reveals two ratios, TP/(TP+FN) and FP/(FP+TN). In other words, ROC reveals true predictions/(true predictions+misses) and false predictions/(false predictions+ correct rejections). Both of the two algorithms were performed on the test datasets of 18 transcription factors. From Table 2, we can see that the AUC of MD-SVM is superior to that of MI-SVM for most of the 18 transcription factors. For example, the AUC of MD-SVM is 0.911275 for Egr2, which is obviously higher than that of MI-SVM. Egr2 (also termed Krox20) is a important transcription regulatory factor for molecular mechanism in gene regulation. It contains two zinc finger DNA-binding sites, and is highly expressed in a population of migrating neural crest cells. In addition, the MD-SVM method has better results on the transcription factor Oct1 than MI-SVM. Previous studies have found that the study of Oct1 transcription factors has important implications for bioinformatics. For example, previous research shows Oct1 is highly polymorphic in ethnically diverse populations. Although most of the results of the MD-SVM algorithm are slightly improved the AUC of the motif discovery, the prediction experiment of transcription factor binding sites is not a simple matter, we need to explore and continuously optimize the results. Although MD-SVM outperforms MI-SVM for most of transcription factors, there are some exceptional TFs such as Pit1. Overall, the results of our new SVM-based algorithm is more reliable than that of existing algorithm in the prediction of transcription factors binding sites.We can observe from the experimental results that although MD-SVM does not greatly improve the accuracy of most transcription factors, our main contribution to the algorithm is the convergence of the algorithm and prevention of over-fitting. Our main improvement is the iterative bounce condition of the algorithm, so that the algorithm can easily complete the iteration in the case of a large amount of data, thereby improving the results of the algorithm. The method of this paper is to use the idea of multi-instance learning in the learning of transcription factor recognition sites, so that it can better model the relationship between transcription factors and DNA.

**Table 2:** Performance comparison between MI-SVM and MD-SVM

Transcription factor	MI-SVM	MD-SVM
Zscan10-3	0.802262	0.802638
Sox14	0.918162	0.918175
Irf2	0.966050	0.966175
Nkx2-9	0.937225	0.937575
Foxg1	0.896850	0.897000
Mlx	0.999125	0.999475
Sdccag8	0.996550	0.996575
Mecp2	0.930225	0.930125
Zfp202	0.913325	0.920475
Egr2	0.899875	0.911275
Dmrtc2	0.968725	0.966925
Pou1f1	0.997725	0.998575
Pou3f1	0.993062	0.993063
Foxo1	0.930800	0.930325
Oct1	0.989450	0.994550
Pit1	0.994875	0.994775
Foxp2	0.925825	0.926425

## Conclusion

With the development of high-throughput technologies, a large amount of sequencing data was generated, such as RNA-seq, PBM and scRNA-seq. It provides an opportunity to understand the molecular mechanism of life processes through computational approaches. Motif discovery for transcription factor binding sites is of central importance in studying DNA-protein interactions, which play major roles in the regulation of gene expressions. However, this problem remains a challenge because of the complexity of binding preference of specific transcription factors. Here, we propose a novel SVM-based MD-SVM, which translate the motif discovery problem into a multiple instance learning model. To evaluate the algorithm performance of MD-SVM and MI-SVM, both of the two algorithms were performed on test datasets of 18 transcription factors, which were commonly used in the DREAM5 challenge. Sequence logos of predicted binding preferences were also compared to that in the database of JASPAR. Results show that our novel MD-SVM algorithm outperforms MI-SVM in terms of both accuracy and precision. The sequence logos of our predicted binding preference are in consistent with these in the JASPAR database. Hopefully, the application of our algorithm in real biological data can help us get a better understanding of molecular regulation and phylogenesis.

## Abbreviations

AUC: Area under the curve
BaMM: Bayesian markov model
MD: Motif discovery
MD-SVM: Motif discovery algorithm based on support vector machine
MI-SVM: Multiple instance learning algorithm based on support vector machine
MIL: Multiple instance learning
PBM: Protein vinding microarray
PWM: Position weight matrix
ROC: Receiver operating characteristic
SVM: Support vector machine
TF: Transcription factor
